# Activating *STAT3* mutations in CD8+ T-cells correlate to serological positivity in rheumatoid arthritis

**DOI:** 10.3389/fimmu.2024.1466276

**Published:** 2024-10-21

**Authors:** Katharine B. Moosic, Thomas L. Olson, Mark Freijat, Samara Khalique, Cait E. Hamele, Bryna Shemo, Jesse Boodoo, William Baker, Gitanjali Khurana, Matthew Schmachtenberg, Tristin Duffy, Aakrosh Ratan, Erika Darrah, Felipe Andrade, Marieke Jones, Kristine C. Olson, David J. Feith, Donald L. Kimpel, Thomas P. Loughran

**Affiliations:** ^1^ University of Virginia Cancer Center, University of Virginia School of Medicine, Charlottesville, VA, United States; ^2^ Division of Hematology/Oncology, Department of Medicine, University of Virginia School of Medicine, Charlottesville, VA, United States; ^3^ Department of Pathology, School of Medicine, University of Virginia, Charlottesville, VA, United States; ^4^ Division of Rheumatology, Department of Medicine, University of Virginia School of Medicine, Charlottesville, VA, United States; ^5^ Center for Public Health Genomics, School of Medicine, University of Virginia, Charlottesville, VA, United States; ^6^ Department of Public Health Sciences, School of Medicine, University of Virginia, Charlottesville, VA, United States; ^7^ Division of Rheumatology, The Johns Hopkins School of Medicine, Baltimore, MD, United States

**Keywords:** rheumatoid arthritis, large granular lymphocytic leukemia, anti-citrullinated protein antibodies, CD8-positive T-lymphocytes, rheumatoid factor, stat3, JAK/STAT

## Abstract

**Objectives:**

Large granular lymphocyte (LGL) leukemia is a rare hematologic malignancy characterized by clonal expansion of cytotoxic T-cells frequent somatic activating *STAT3* mutations. Based on the disease overlap between LGL leukemia rheumatoid arthritis (RA)a putative role for CD8+ T-cells in RA we hypothesized that *STAT3* mutations may be detected in RA patient CD8+ T-cells correlate with clinical characteristics.

**Methods:**

Blood samples, clinical parameters, and demographics were collected from 98 RA patients and 9 healthy controls (HCs). CD8+ cell DNA was isolated and analyzed via droplet digital (dd)PCR to detect *STAT3* mutations common in LGL leukemia: Y640F, D661Y, and the S614 to G618 region. *STAT3* data from 99 HCs from a public dataset supplemented our 9 HCs.

**Results:**

RA patients had significantly increased presence of *STAT3* mutations compared to controls (Y640F p=0.0005, D661Y p=0.0005). The majority of these were low variant allele frequency (VAF) (0.008-0.05%) mutations detected in a higher proportion of the RA population (31/98 Y640F, 17/98 D661Y) vs. HCs (0/108 Y640F, 0/108 D661Y). In addition, 3/98 RA patients had a *STAT3* mutation at a VAF >5% compared to 0/108 controls. Serological markers, RF and anti-CCP positivity, were more frequently positive in RA patients with *STAT3* mutation relative to those without (88% vs 59% RF, p=0.047; 92% vs 58% anti-CCP, p=0.031, respectively).

**Conclusions:**

*STAT3* activating mutations were detected in RA patient CD8+ cells and associated with seropositivity. Thus, *STAT3* activating mutations may play a role in disease pathogenesis in a subset of RA patients.

## 1 Introduction

Rheumatoid arthritis (RA) is a chronic systemic autoimmune disease that can lead to joint inflammation, erosion, and pain, as well as disability and shortened life expectancy. Diagnosis is determined by symptomatic presentation and duration, and aided by the presence of rheumatoid factor (RF) and/or anti-cyclic citrullinated peptide (CCP) positivity (1, 2). RF is indicative of chronic antigenic stimulation and may be present in a variety of inflammatory disease processes. In the context of RA, it is generally predictive of more aggressive disease and severe erosions. Anti-CCP autoantibodies are more specific to RA and even more predictive of erosive disease than RF (1, 3).

Large granular lymphocyte (LGL) leukemia is a rare hematologic malignancy characterized by clonal proliferation of natural killer or CD8+ T-cells and findings like neutropenia and anemia. Interestingly, LGL leukemia is strongly associated with multiple autoimmune diseases, with as high as 36% of patients exhibiting concomitant RA (4, 5). There is significant overlap between these diseases, with both RA alone and in combination with LGL leukemia exhibiting cytotoxic T-cell expansions, HLA-DR4 enrichment, female bias, RA-associated autoantibodies, and similar treatment responses (e.g., methotrexate) (6).

The Janus kinase (JAK)/signaling transducer and activator of transcription (STAT) signaling axis is commonly dysregulated in both RA and LGL leukemia (7, 8). The principal molecular hallmark of LGL leukemia is somatic activating *STAT3* mutations that may drive CD8+ T-cell expansion. The prevalence of *STAT3* mutations in LGL leukemia ranges from 27-72% depending on the cohort, but most patients exhibit increased activation of *STAT3* through phosphorylation regardless of *STAT3* mutational status (9). The SH2 domain, which mediates STAT3 dimerization, is frequently mutated, with Y640F and D661Y mutations accounting for ~70% of those identified (10, 11). The S614-G618 region of the SH2 domain is also commonly mutated in LGL leukemia patients (12, 13). Of note, LGL leukemia patients with multiple *STAT3* mutations are more likely to experience concomitant RA (14). *STAT3* mutations are also associated with more favorable response to methotrexate treatment (15).

*STAT3* mutations are also found in a variety of cell types across both autoimmune diseases and hematologic disorders such as myelodysplastic syndrome, aplastic anemia, celiac disease, and multiple sclerosis (16–18). Felty Syndrome, a subtype of RA characterized by symptoms similar to LGL leukemia such as concomitant neutropenia and splenomegaly, demonstrates a *STAT3* mutation rate of 43% (19).

Based on these observations, we hypothesized that *STAT3* mutations may be prevalent in CD8+ T-cells of RA patients, and the presence of mutations may correlate with specific clinical characteristics.

## 2 Materials and methods

### 2.1 Patient selection

Research was conducted under a protocol approved by the University of Virginia (UVA) institutional review board (study #18519), and informed consent was obtained prior to conducting any study-related procedures. The first 150 patients seen at UVA rheumatology meeting classification criteria for RA as defined by the American College of Rheumatology (ACR) or European League Against Rheumatism (EULAR) (2, 20) were selected. Exclusion criteria included prisoner status, non-english speaking, anemia with hemoglobin <10g/dL, and pregnancy. The 150 participant number was targeted to maintain adequate sample size post processing based on prior power calculations.

### 2.2 Clinical data

Medical records were reviewed using UVA’s EPIC EMR system. Outside records were requested in CareEverywhere through the UVA EPIC system. Demographics represent the most recent data available at the time of data mining.

Date-dependent clinical parameters were collected on or as close to the date of research sample collection as possible. In cases where laboratory results or other data for the same test existed in approximately the same timeframe both before and after research sample collection, results obtained on the date preceding sample collection were chosen. Raw values and positivity status were collected for each parameter, with positivity evaluation based on laboratory clinical standards as noted for each patient.

Clinical data acquired from multiple physicians, laboratories, and hospitals were harmonized into a uniform format. Either Disease Activity Score in 28 joints (DAS28) or Clinical Disease Activity Index (CDAI; whichever was available) was recorded for each patient, and values were converted to standard scores of remission, low, moderate, and high disease activity (</= 2.8, 2.9-10, 10.1-22, >22 for CDAI; <2.6, 2.6-3.0, 3.1-5.0, >5.0 for DAS28). Double positivity refers to patients with available data for both RF and historical anti-CCP and both were positive.

Complete blood counts, erythrocyte sedimentation rate (ESR), and C-reactive protein (CRP) values are routine tests for ongoing RA management, so most were on or near the date of research sample collection. RF and anti-CCP testing are used diagnostically and thus collected less frequently, so were often found at dates farther from sample collection. As RF and anti-CCP values tend to be less labile, this increased gap between clinical test and research sample for RF and anti-CCP was deemed allowable. Of note, these values mined from patient charts are denoted as historical anti-CCP. Current anti-CCP values were derived from sera collected at the same timepoint as that used for ddPCR analysis and determined with the QUANTA Lite^®^ CCP3.1 IgG/IgA ELISA kit (positivity cutoff ≥20 U/mL).

### 2.3 Sample preparation and CD8+ isolation

Fresh peripheral blood mononuclear cells (PBMCs) from 150 RA patients and 9 HCs (Research Blood Components) were isolated using Ficoll-Paque gradient separation per manufacturer’s instructions (Cytiva). Then fresh CD8+ T-cells were isolated using RosetteSep reagent according to manufacturer’s instructions (StemCell Technologies). Genomic DNA (gDNA) was extracted from isolated CD8+ cells using the Anaprep Cultured Cell DNA Extraction Kit (Biochain) and stored at -20°C.

### 2.4 Droplet digital PCR *STAT3* mutation analysis

gDNA from isolated CD8+ T-cells was not sufficient to allow replicates of multiple assays; therefore, gDNA was pre-amplified as a single amplicon suitable as input for all downstream assays. Samples were tested with a quantification assay to determine starting copy number, and 40,000 genomic copies were used as input for pre-amplification. After amplification, the D661 wild-type (WT) assay was performed to quantify pre-amplification outputs. 40,000 amplified copies were then used as input for the D661Y and Y640F mutation-specific assays and the S614-G618 dropout assay designed to detect any mutation in that region. ddPCR was performed using the QX200 and digital droplet generator, ddPCR Supermix for probes (No dUTP), and FAM/HEX probes under manufacturer’s recommendations (BioRad) (**Supplementary Figure 1**). The three samples with the highest frequency mutations were confirmed by next-generation sequencing as previously described (21).

At least two separate runs using 40,000 amplified copies as input were performed for each mutation-specific assay. A third confirmatory assay was performed using 20,000 amplified copies as input. Biorad’s Quantasoft software provided the variant allele frequencies (VAFs) based on a Poisson distribution, and thresholding was placed manually based on the overlay of all samples on each plate with a threshold cutoff of at least 3 positive droplets per well as per manufacturer recommendations. Sample VAFs were considered replicated and used for statistical analysis if they had at least two runs with VAFs in the same bin (Bins: <threshold, 0.008-0.05%, >0.05-5%, >5%). Assuming heterozygosity, the maximum bin cutoff is equivalent to 1 mutation in every 10 cells, and the minimum threshold is equivalent to 1 mutation in every 6,250 cells. Following all pre-amplification and ddPCR assay steps, 98 patients had sufficient sample volume for analysis.

### 2.5 Statistical analysis

Statistical testing was performed in R (ver 4.3.0). Chi-squared tests with p-values based on Monte Carlo simulation were used for categorical variables, comparing different VAF bins, WT to *STAT3* mutant patients, and Y640F patients to D661Y. ANOVA compared clinical features between WT, D661Y, and Y640F groups based on mutation status. Welch’s ANOVA or Kruskal-Wallis was used depending on assumptions. T-tests compared quantitative variables between WT and *STAT3* mutant samples. Either Welch’s T or Wilcoxon Rank Sum tests were used depending on assumptions. All tests and p-values are in **Supplementary Tables 1**-**3**. The cutoff for p*-*value significance was <0.05. Sample sizes varied based on clinical data availability. Adjustment of p-values for multiple comparisons was done for each test type using the Holm method (**Supplementary Tables 1**-**3**). Sensitivity analyses investigating clinical differences between patients with or without *STAT3* mutation were performed using either a cutoff VAF≥0.008% or VAF>0.05%, with the testing from VAF>0.05% reported in all tables and figures except **Supplementary Figure 2** which uses ≥0.008% as the cutoff.

We modeled mutation presence (VAF≥0.008%) as a function of RA status and age using logistic regression. The model incorporated data from our ddPCR analysis as well as from a published dataset of 99 HCs that utilized deep amplicon sequencing of the two main exons (20 and 21) of the *STAT3* SH2 domain, with 2x300bp reads optimized for high sequencing depth (>25,000x). Their resulting VAF range (0.007%-1.2%) was comparable to the 0.008%-5.93% we observed using our targeted ddPCR assays (22).

## 3 Results

### 3.1 *STAT3* activating mutations are detected in RA patient samples

**Figure 1:**
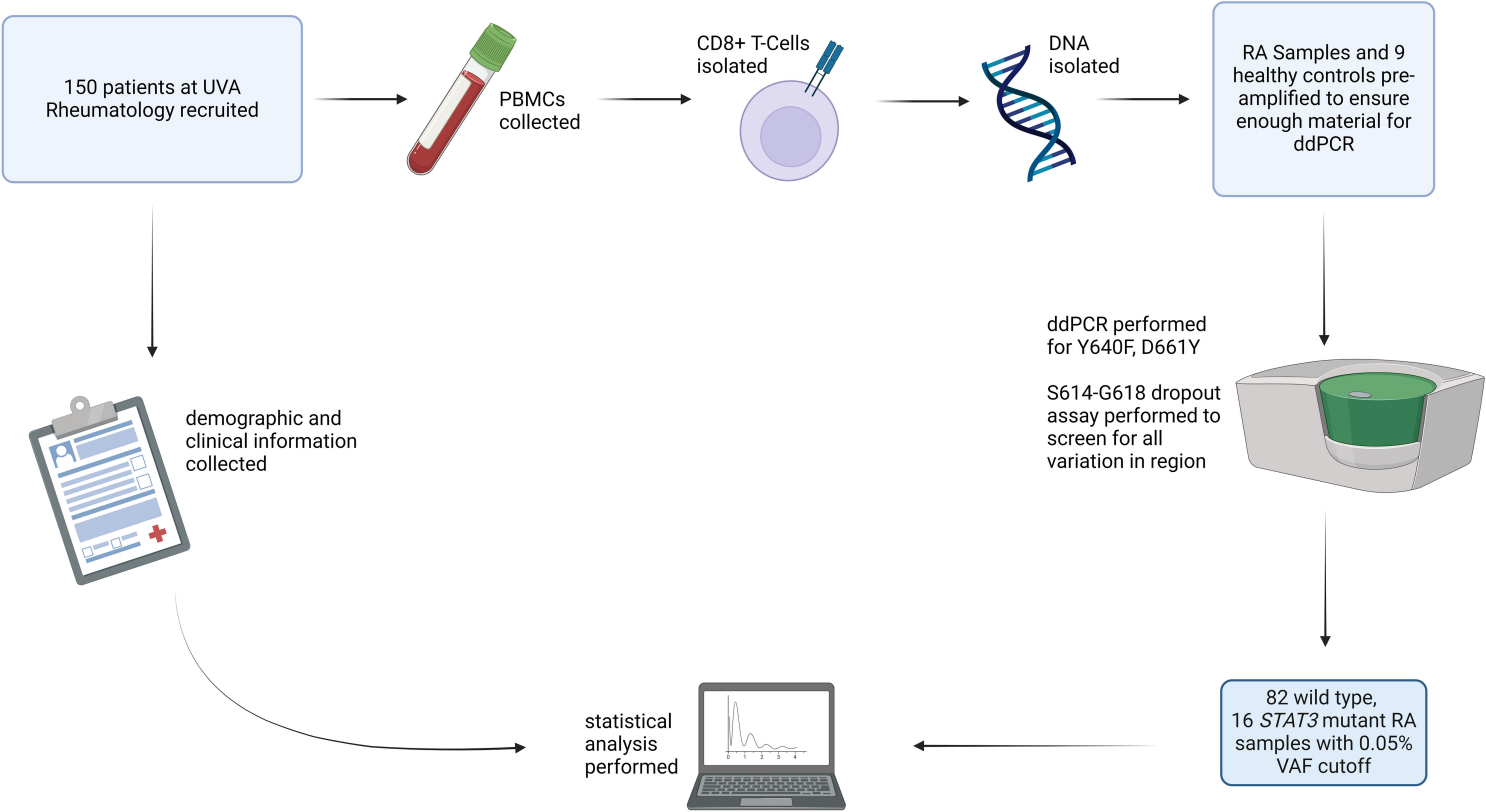
Study overview: RA patients (n=150) were recruited for the study, and blood samples from HCs (n=9) were obtained. PBMCs were isolated from these samples. CD8+ T-cells were then isolated, DNA was extracted, and each sample was assayed using ddPCR for *STAT3* Y640F and D661Y mutations as well as mutations in the S614-G618 region. After all processing and analysis was completed, 98 samples had sufficient material to determine mutation presence. Using a cutoff VAF of 0.05%, 82 were identified as WT and 16 as mutant *STAT3*. RA patient mutation status was then correlated with clinical parameters. Created with Biorender.com.

**Figure 2:**
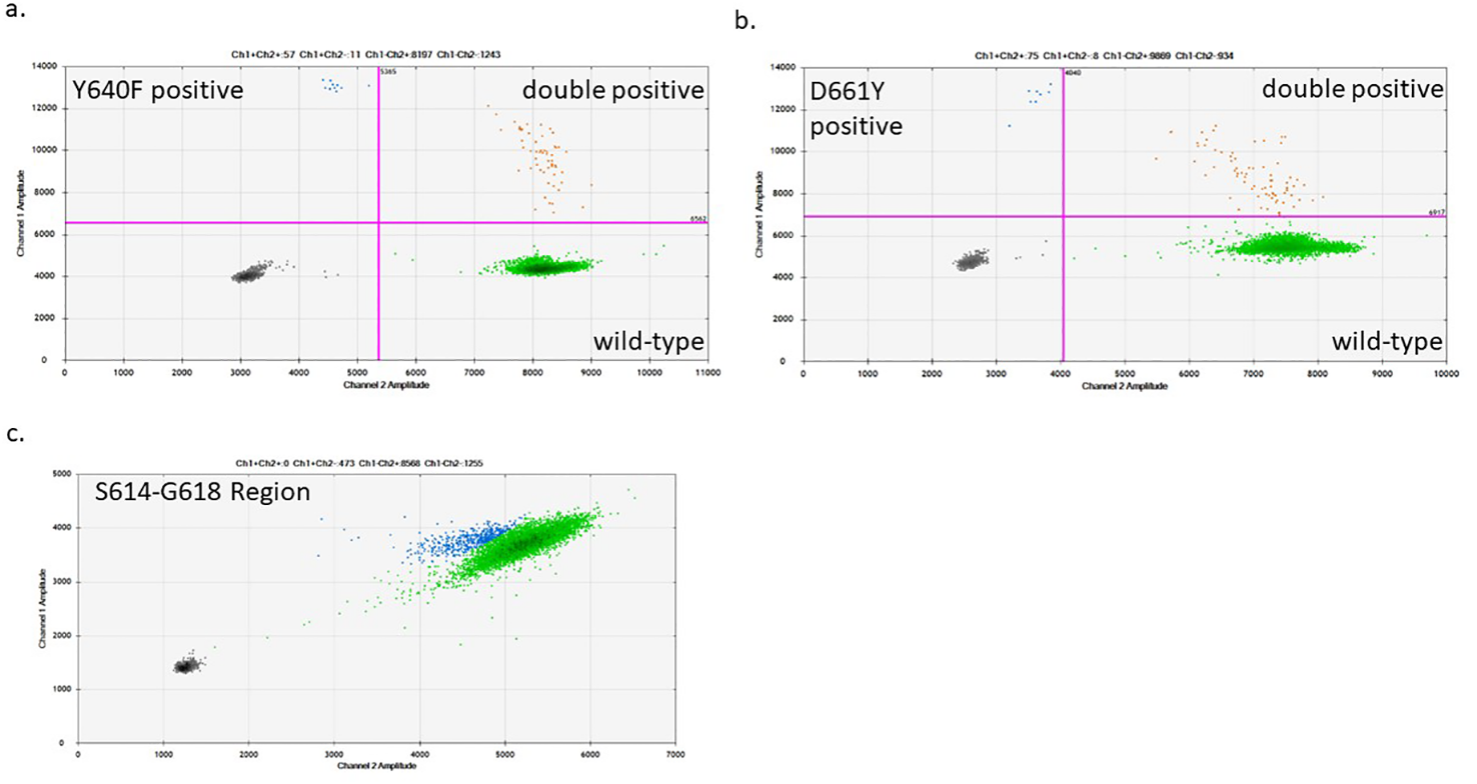
Sample ddPCR plots of *STAT3* mutant detection in CD8+ T-cells: The *STAT3* mutant region was pre-amplified from an input of 40,000 genomic copies. After the pre-amplification step, 40,000 amplified copies were used for the D661Y and Y640F mutation-specific ddPCR assays **(A, B)** and the S614-G618 region dropout assay **(C)**. **(A, B)** The bottom right quadrant (green) shows droplets containing the WT product, the top left quadrant (blue) shows those with the mutant product, and the top right quadrant (orange) shows droplets with both WT and mutant DNA detected in the same droplet. **(C)** The S614-G618 assay was designed as a dropout assay where the mutant-detecting probe spans the entire region encoding for this range of amino acids, while an adjacent WT probe detects which droplets contain DNA. Green droplets have full intensity for each probe (WT), while blue droplets are those with diminished intensity indicating that a mutation exists in the probe-binding region.

Due to the putative role of cytotoxic T-cells in RA, symptomatic overlap, and the frequent co-occurrence of LGL leukemia with RA (6, 23), we hypothesized that CD8+ T-cells from RA patients may exhibit a higher prevalence of *STAT3* mutations than healthy individuals. To address this question, we isolated CD8+ cells from the whole blood of RA patients, extracted, and then amplified DNA from these cells. The DNA was assayed by ddPCR to detect the following *STAT3* mutations: Y640F, D661Y, or any mutations in the region of S614-G618 of *STAT3* (**Figure 1**). Each assay showed distinct regions of positive droplets that were used to determine VAF (**Figure 2**). Example ddPCR outputs of zero, near threshold, and high VAF are shown in **Supplementary Figure 3**. Y640F and D661Y ddPCR assays utilized highly sensitive allelic discrimination assays as previously reported (12). The S614-G618 assay was designed to detect any mutation in that region. As a dropout assay, less sensitivity is expected as separation of mutant from WT droplets is not as distinct (**Figure 2**).

*STAT3* ddPCR VAFs were determined for 98 RA patients and 9 HCs (**Table 1**). Overall, we observed that 52/98 RA patients had either Y640F or D661Y mutations (VAF ≥0.008%), significantly more than 1/9 in HCs (p=0.029, Chi-Squared test). The resulting VAFs were divided into four different ranges, <threshold, 0.008-0.05%, >0.05%-5%, and >5%. Of note, no mutations were detected between 1% and 5% VAF. The upper bin was set at >5% to represent a minimum 1:10 cells with a mutation assuming heterozygosity.

**Table 1:** *STAT3* mutations detected by ddPCR analysis of CD8+ T-cell DNA.

Healthy Control Samples	<Threshold	0.008%-0.05% VAF	>0.05%-5% VAF	>5% VAF
Y640F	8/9	0	1/9	0
D661Y	9/9	0	0	0
S614-G618 Region	n/a	n/a	n/a	0
RA Samples	<Threshold	0.008%-0.05% VAF	>0.05%-5% VAF	>5% VAF
Y640F	57/98	31/98	9/98	1/98
D661Y	73/98	17/98	8/98	0
S614-G618 Region	n/a	n/a	n/a	2/98

Patient and HC samples were analyzed via ddPCR. The *STAT3* VAF for each sample was automatically calculated by Quantasoft software after manually thresholding each sample’s ddPCR output plot. The difference in mutation distribution between HCs and RA samples was assessed using Pearson’s Chi-Square test for both Y640F and D661Y mutations (p=0.106, p=0.264 respectively). The <Threshold category contains any samples with fewer than three droplets (rule of three). The S614-G618 data is based on a dropout assay with less sensitivity than the Y640F and D661Y assays. Displayed VAFs for the S614-G618 assay are based on confirmatory rhAmpSeq values.

The majority of HCs (8/9 Y640F assay, 9/9 D661Y assay) had VAFs below the 3-droplet threshold of the assay. Of the RA patients sampled, 3.1% (3/98) had VAFs above 5% for any of the three mutations analyzed, and 10/98 Y640F and 8/98 D661Y mutant samples had *STAT3* mutations above 0.05% VAF. Y640F and D661Y mutations were frequently detected in the RA samples in the 0.008-0.05% VAF range (Y640F 31/98 and D661Y 17/98), but not at all in the HCs (Y640F 0/9 and D661Y 0/9). Several RA patients had detectable levels of both mutations (14/98). Interestingly, 7/8 of the highest VAF (>0.05%) D661Y patients also had some level of Y640F mutation. Of note, the two mutations detected with the S614-G618 dropout assay were confirmed via rhAmpSeq, and the variants were S614R and G618R, both of which have been previously reported in LGL leukemia (12, 24).

### 3.2 Confirmation of *STAT3* mutation differences between RA and healthy donors

**Figure 3:**
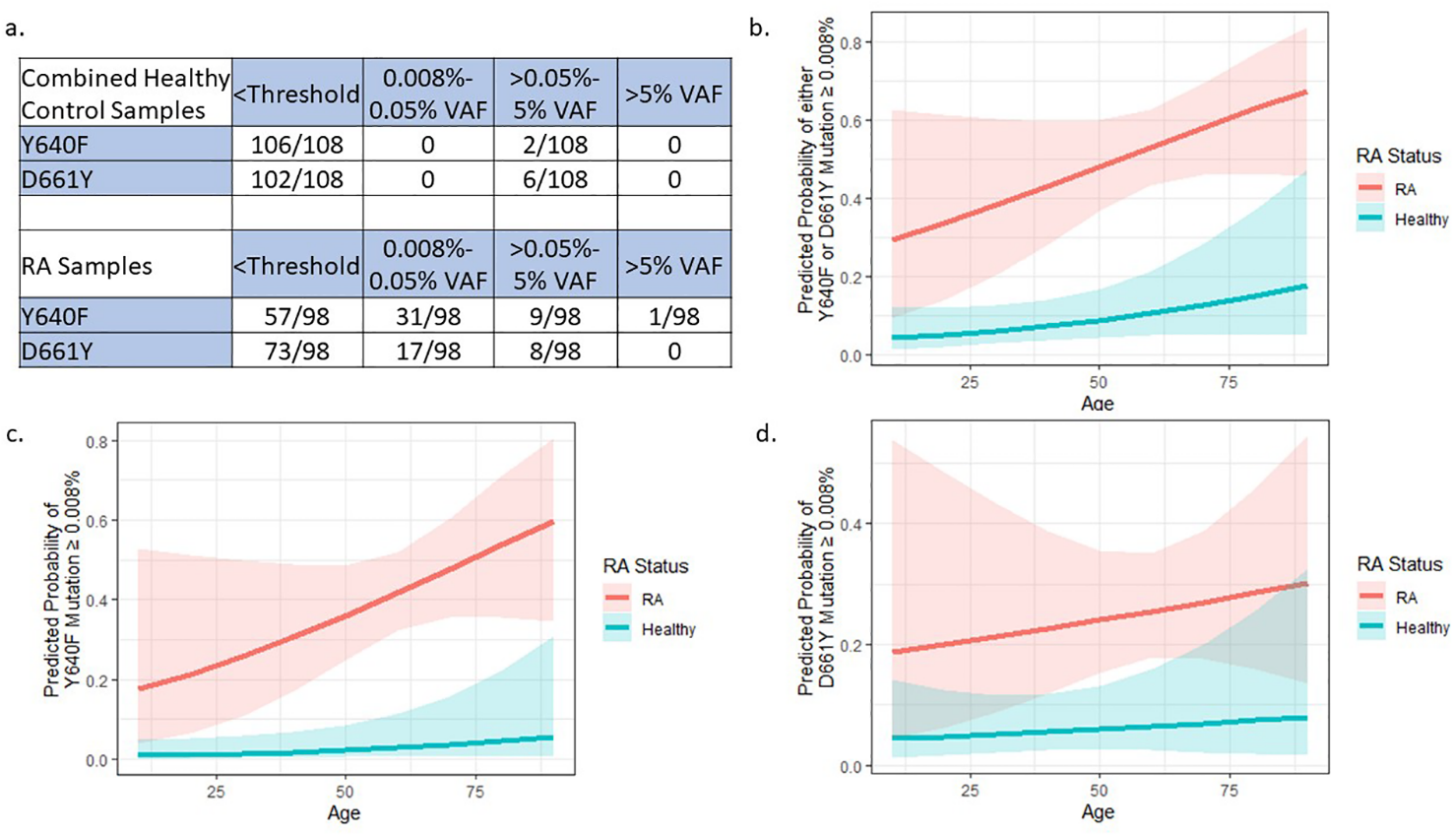
External dataset confirms *STAT3* mutation differences between RA and HCs: **(A)** Data from 99 HC samples (22) were added to our 9 HCs to give an expanded sample size of 108. The 98 RA samples were only from our current study and are the same as **Table 2** (no RA samples were assessed in the Valori et al. study). Compared to RA samples, the combined HCs have statistically lower frequencies of Y640F and D661Y mutations (p=0.0005 and p=0.0005, respectively) by Pearson’s Chi-Squared test. **(B–D)** Logistic regression models to explain the presence of *STAT3* mutations using age and RA status were built by binning mutation presence at a VAF cutoff of 0.008% (3 or more droplets detected). These models includes HC data from Valori, et al (22). RA patients were more likely to have **(B)** any mutation either Y640F or D661Y, **(C)** only Y640F, or **(D)** only D661Y mutations compared to HCs (p=1.74x10^-6^, p=4.57x10^-5^, p=0.005 respectively). Graphs **(B–D)** created in R.

To validate this difference in mutation presence between RA samples and HCs, we utilized a publicly available dataset from Valori, et al. (22) This study utilized deep amplicon sequencing of the two main exons of the *STAT3* SH2 domain to determine the prevalence of *STAT3* mutations in healthy donor CD8+ T-cell DNA with nearly identical sensitivity to our ddPCR assay (**Figure 3A**). In their screen for mutations across the *STAT3* SH2 domain, Valori et al. observed that 24/99 controls exhibited any mutation. However, only 7/99 (VAF 0.07-0.18%) had Y640F or D661Y mutations, with only 1/99 having a Y640F substitution. The prevalence of either Y640F or D661Y mutations in this larger control population did not differ significantly from our control dataset (p=0.17 and p=1, respectively).

Comparison of the Y640F and D661Y *STAT3* distribution of this new cohort of 108 healthy donor samples (99 individuals plus 9 from our study), to the 98 RA patients in our study showed a statistically different VAF distribution of *STAT3* mutations in RA patients compared to HCs (Y640F mutation p=0.0005 and D661Y mutation p=0.0005, Pearson’s Chi-Squared test), with the majority of the difference stemming from mutations in the 0.008%-0.05% VAF range (**Figure 3A**).

To account for the potential confounding factor of patient age differences between cohorts, logistic regression was used to explain the presence of any detectable mutation (≥0.008% VAF) using RA status and age. Even after taking age into account, there was a significantly higher probability of *STAT3* mutation in RA patients compared to HCs across both mutation types (Either Y640F or D661Y p=1.74x10^-6^, Y640F alone p=4.57x10^-5^, D661Y alone p=0.00465) (**Figures 3B–D**).

### 3.3 Clinical features of *STAT3* WT and mutant patients

In LGL leukemia, *STAT3* mutations are correlated to specific disease phenotypes and response to treatment (11, 13, 15, 25). Therefore, we investigated the association of *STAT3* mutations with clinical and demographic characteristics of RA patients to determine whether *STAT3* mutation presence was related to disease manifestation. Many of the mutations detected in this study are in a very small proportion of CD8+ T-cells. Initially we looked at the clinical differences between WT patients and those with any detectable *STAT3* mutation (VAF≥0.008%) and observed no strong differences between groups (p-values in **Supplementary Tables 1**-**3**). With the assumption that there may be a minimal level of mutation necessary to impact clinical features, we used a higher cutoff (VAF >0.05%, equivalent to 1:1000 heterozygous mutant cells) that captured approximately the upper quartile of all mutations. All samples with a VAF >0.05% for any of the three mutation assays were considered *STAT3* mutant (n=16), and the rest were considered WT (n=82). Four main categories were examined: patient characteristics, measures of disease state, hematologic parameters, and treatment (**Table 2**).

**Table 2:** Clinical features of the RA patient cohort.

	WT	All *STAT3* Mutations
Patient Characteristics		
Number of Patients	82	16
Age at Sampling in years (range)	61 (26-85)	62 (39-77)
Sex		
Female (%)	62/82 (76)	10/16 (63)
Male (%)	20/82 (24)	6/16 (38)
Race		
White (%)	67/82 (82)	16/16 (100)
African American/Black (%)	13/82 (16)	0/16 (0)
Asian (%)	2/82 (2)	0/16 (0)
Time since dx in years (range)*	6 (0-56)	10 (3-32)
Measures of Disease State		
Erosive disease (%)	49/81 (60)	8/16 (50)
CDAI/DAS28 Descriptive Scores		
Remission (%)	25/76 (33)	4/16 (25)
Low (%)	28/76 (37)	6/16 (38)
Moderate (%)	16/76 (21)	5/16 (31)
High (%)	7/76 (9)	1/16 (6)
CDAI/DAS28 Component Scores (range)		
Swollen Joint	0 (0-26)	0 (0-9)
Tender Joint	0 (0-26)	0 (0-13)
Patient Global	3 (0-70)	4 (0-50)
Provider Global	2 (0-8)	1 (0-10)
CRP in mg/L (range)	0.4 (0-45.4)	0.35 (0-3.1)
CRP Positivity (%)	2/82 (2)	0/16 (0)
ESR in mm/hr (range)	25 (0-116)	26 (3-43)
ESR Positivity (%)	36/82 (44)	6/15 (40)
Hematologic Parameters in K/µL (Range)		
White Blood Cell Count (Ref: 4.5-11)	7.05 (3.32-41.15)	6.58 (3.03-10.50)
Absolute Lymphocyte Count (Ref: 1-4.8)	1.73 (0.26-3.88)	1.59 (0.86-2.60)
Absolute Neutrophil Count (Ref: 1.5-8)	4.14 (1.22-38.93)	3.64 (0.96-9.12)
Treatment Information		
# of Treatments (range)	3 (0-10)	3 (1-10)
Methotrexate Exposure (%)	70/82 (85)	14/16 (88)
TNF Inhibitor Exposure (%)	35/82 (43)	8/16 (50)

Patients are stratified as WT or STAT3 mutant (VAF >0.05%). Clinical features were collected at the time of enrollment, so information is matched to the time of mutant detection by ddPCR, excluding parameters only measured at diagnosis like RF and anti-CCP. Median values are listed for each parameter. The ranges listed next to the hematological parameters are reference ranges. Statistical significance between groups is denoted by an asterisk. All p-values and statistical tests used listed in **Supplementary Tables 1**, **2**.

The overall cohort exhibited similar demographics to what had previously been reported for RA. Clinical features were similar between mutant and WT groups for most parameters with exceptions such as time since diagnosis graphed in **Supplementary Figure 4**. While 59% (57/97) of patients exhibited erosive disease, the majority of patients with available data had well-controlled disease, as indicated by CDAI/DAS28 values in the remission and mild disease categories for 67% (62/92) of individuals. CRP serum levels differed across WT, Y640F, and D661Y, but the average values (1.67 mg/L, 0.29 mg/L, 1.12 mg/L respectively, p=0.04 Welch’s ANOVA) in each category were well below the positivity cutoff of 10 mg/L (**Supplementary Figure 4**). Blood counts were typically in the normal range, and most patients were exposed to methotrexate (86%, 84/98), while <50% were exposed to TNF-α inhibitors.

### 3.4 Patients with *STAT3* mutations exhibit serological positivity

**Figure 4:**
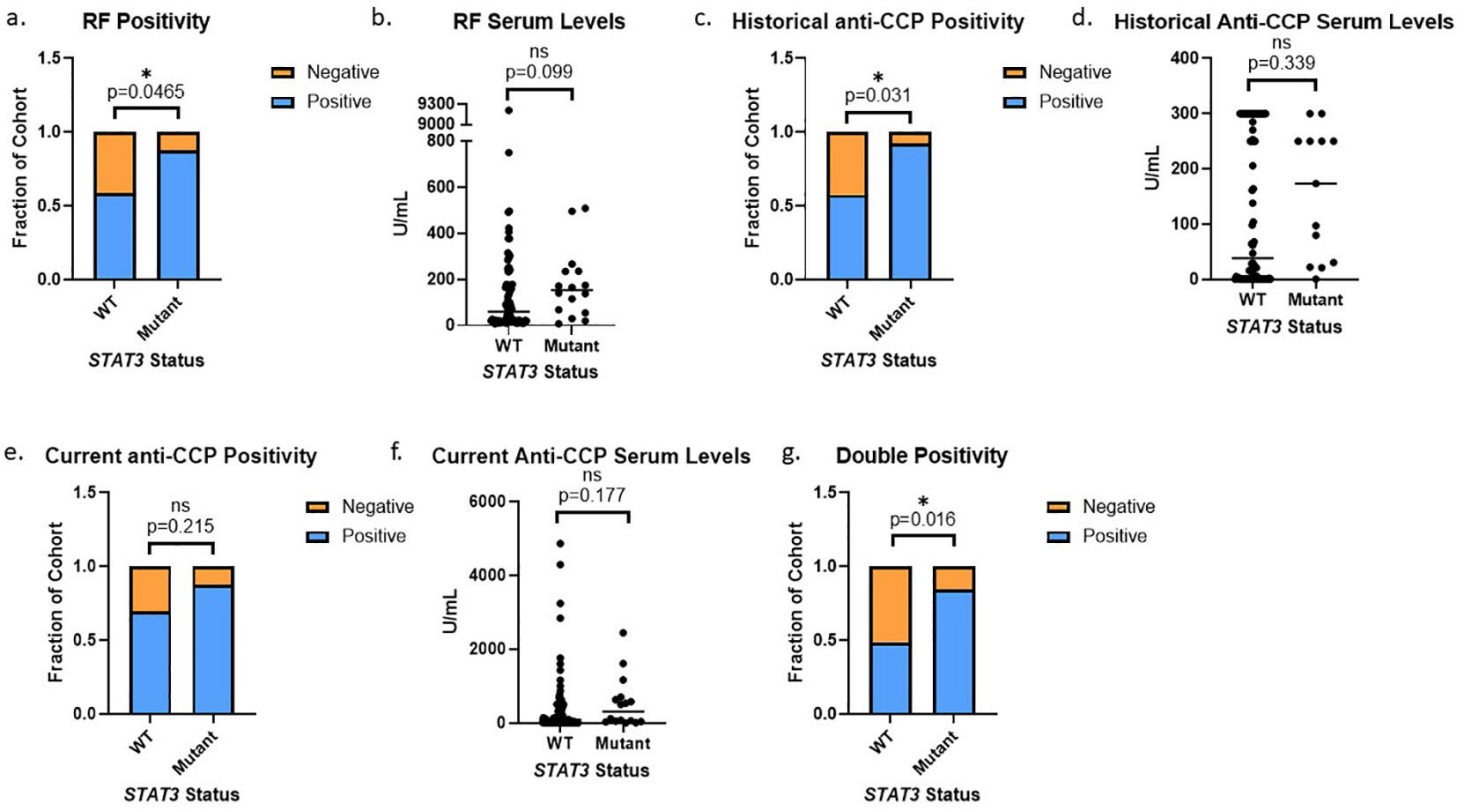
*STAT3* mutation correlates to seropositivity in RA patients: **(A)** RA patients with *STAT3* mutations exhibited more RF positivity than WT patients (14/16, 88% vs 48/82, 59%, p=0.047 Pearson’s Chi-Squared). **(B)** RA patients with *STAT3* mutations also had higher median serum RF values (152.5 U/mL vs 59.6 U/mL, p=0.099 Wilcoxon Test). **(C)** RA patients with *STAT3* mutations exhibited more historical anti-CCP positivity than WT patients (12/13, 92% vs 46/80, 58% p=0.031 Pearson’s Chi-Squared). **(D)** RA patients with *STAT3* mutations also had higher median anti-CCP values (173.5 U/mL vs 38.5 U/mL, p=0.339 Wilcoxon Test). **(E)** Following the same trend, the *STAT3* mutant group exhibited slightly more current anti-CCP positivity (p=0.215) and **(F)** also had higher median anti-CCP values (305.7 U/mL vs 94.3 U/mL, p=0.177 Wilcoxon Test). **(G)** RA patients with *STAT3* mutations exhibited more double positivity for both RF and anti-CCP than WT patients (11/13, 85% vs 39/80, 49% p=0.016 Pearson’s Chi-Squared). All graphs created in prism. Adjusted p-values for each statistical test are available in **Supplementary Tables 1**-**3**. Star denotes statistical significance.

Utilizing a threshold cutoff of any detectable mutation (VAF ≥0.008%) for comparison across WT and *STAT3* mutant groups, we observed a correlation between the presence of *STAT3* mutation and seropositivity for either RF or anti-CCP (**Supplementary Figure 2**). The majority of patient samples in the >0.05% VAF bin were above the threshold for seropositivity (**Supplementary Figure 5**). Using this refined 0.05% cutoff, we observed that seropositivity rates were significantly higher in patients with *STAT3* mutations vs WT. Specifically, RF positivity was significantly more prevalent (p=0.047) in patients with mutant *STAT3* (87.5%) than WT *STAT3* (58.5%) (**Figure 4A**), with the median value of RF in patients with mutant *STAT3* (Median: 152.5 U/mL, Q1: 64.6 U/mL, Q3: 234.3 U/mL) trending higher compared to WT *STAT3* (Median: 59.6 U/mL, Q1: 20 U/mL, Q3: 176.8 U/mL, p=0.099) (**Figure 4B**). Historical anti-CCP positivity was also significantly more common (p=0.031) in patients with mutant *STAT3* (92.3%) compared to WT *STAT3* (57.5%) (**Figure 4C**). Anti-CCP levels trended higher (p=0.339) in the *STAT3* mutant group (Median: 173.5 U/mL, Q1: 30.4 U/mL, Q3: 250 U/mL) compared to WT (Median: 38.5 U/mL Q1: 0.5 U/mL, Q3: 300 U/mL), but due to the lack of titration in clinical testing, the full potential range of serum levels was not represented in the data available (**Figure 4D**). Current anti-CCP levels measured from the same time point as sequencing were completed in-house to standardize and determine the full range of serum values in patients. Both the current anti-CCP positivity (*STAT3* mutant 87.5% vs. WT 69.5%, p=0.216) and levels (*STAT3* mutant Median: 305.7 U/mL, Q1: 47 U/mL, Q3: 649 U/mL vs. WT Median: 94.3 U/mL, Q1: 9.6 U/mL, Q3: 456.3 U/mL p=0.177) exhibited similar trends to the historical values but lacked significance (**Figures 4E, F**). However, double positivity for RF and anti-CCP was significantly more common (p=0.016) in mutant *STAT3* (84.6%) vs. WT RA patients (48.8%) (**Figure 4G**).

## 4 Discussion

This is the first study to utilize ddPCR analysis to identify *STAT3* somatic activating mutations in CD8+ T-cells of RA patients. There were significantly more *STAT3* mutations in RA patients (52/98) compared to HCs (1/9). We observed that 3.1% of subjects with established RA exhibited *STAT3* mutations large enough to indicate CD8+ T-cell clonal expansion. There were far more mid-range (0.008-0.05% VAF) mutations in RA patients (32% Y640F and 17% D661Y) compared to HCs (0%). RA patients with *STAT3* mutations were more likely to be positive for RF and anti-CCP, key diagnostic markers of RA. We also showed for the first time that the presence of activating *STAT3* mutations in CD8+ T-cells is a novel commonality between RA and LGL leukemia. This adds to the existing commonalities which include symptomatic and treatment overlap, reliance on similar signaling pathways such as JAK/STAT signaling, and cytotoxic T-cell expansions.

In LGL leukemia patients, RA is a frequent comorbidity (6). LGL leukemia is typically an indolent disease only requiring treatment if symptomatic. Methotrexate is the most common first-line treatment for both LGL leukemia and RA patients (26, 27), with >85% of patients in our cohort using methotrexate at some point during their disease course. Therefore, we hypothesize that methotrexate treatment to control RA may suppress the expansion of clonal CD8+ T-cells and lead to potential underdiagnosis of LGL leukemia in RA. Indeed, Schwaneck et al. observed that 3.6% of their 529 RA patient cohort had clonal cytotoxic T-cell expansions (28). We observed a similar rate of *STAT3* mutations in CD8+ T-cells at levels high enough to be considered clonal, i.e. 3/98 (3.1%) patients had a VAF >5%. With the assumption that detected mutations are heterozygous, greater than 10% of CD8+ T-cells may harbor the same mutation. Additionally, ddPCR detects only specific nucleotide changes, and there are many other reported germline and somatic *STAT3* activating mutations (11, 29). Massively parallel sequencing of the entire *STAT3* coding region may identify RA patients harboring *STAT3* mutations other than the most commonly occurring variants that were screened in this study. Although we did not verify that the detected mutations were somatic, germline mutations would be expected at VAFs closer to 50% and are associated with autoimmune and lymphoproliferative manifestations not seen in our RA cohort (29). This implies a high likelihood that these mutations are specific to the blood compartment, if not entirely confined to the CD8+ T-cell pool. The frequent observance of these STAT3 mutations in CD8+ T-cells may also be interpreted as a precursor condition to LGL leukemia with as of yet undefined mechanisms required for progression to true LGL leukemia. Longitudinal monitoring in a large cohort of STAT3 mutant RA patients would provide more insight into the relationship between *STAT3*, RA, and LGL leukemia.

Although both Felty Syndrome and LGL leukemia patients with concomitant RA have been reported to display *STAT3* mutations (6, 19, 30), this is the first time that *STAT3* mutations have been reported in a cohort of standard RA patients. One limitation of our study was the number of healthy donor samples and the potential that STAT3 is recurrently mutated at low frequencies in the general population. However, a study of lymphoid clonal hematopoiesis in PBMCs of 46,000 healthy individuals sequenced a panel of frequently mutated genes related to lymphoid malignancies and showed just four individuals (0.00008%) had detectable mutations in *STAT3* (31). Thus, *STAT3* mutations are exceedingly rare in PBMCs of healthy populations, implying that the increased frequency of *STAT3* mutations in our RA cohort is disease-specific. Indeed, we observed 3/98 (3%) RA samples with *STAT3* mutations that meet the traditional threshold of ≥2% VAF utilized for variant calls of mutated genes in myeloid clonal hematopoiesis (31), and we observed many more at even lower frequencies. Additionally, the prevalence of clonal hematopoiesis in RA is similar to that of the general population, suggesting that the increased rates of *STAT3* mutations are not due to increased overall frequencies of mutations in RA (32). By isolating CD8+ T-cells and utilizing ddPCR, our study had a refined ability to detect these rare *STAT3* mutations. Combination of our results with the Valori et al. cohort which also isolated CD8+ T-cells and utilized a technique with high sequencing depth (22), further strengthened our observation that the detection of *STAT3* mutations is specific to RA. Additionally, using the data from Valori et al. in a logistic regression model, we were able to demonstrate that RA patients are significantly more likely to have a mutation in *STAT3* regardless of age (**Figures 3B–D**).

In addition to detection of *STAT3* mutations, this study determined that like LGL leukemia, the presence of *STAT3* mutations correlated with clinical features in RA. We observed that RF and anti-CCP positivity, the two main diagnostic markers used for RA, were both associated with *STAT3* mutations. Although *STAT3* mutation has not yet been correlated to RF and anti-CCP in LGL leukemia, LGL leukemia patients with concomitant RA have higher levels of both RF and anti-CCP than general RA populations, with one study observing 88% anti-CCP positivity and 82% RF positivity (30) and another showing 95% anti-CCP positivity (33). Even without RA, LGL leukemia patients often exhibit positivity for RF, with most frequencies reported at 39-61% (one Chinese cohort reports 10%) (34). Perhaps these increased rates of RF and anti-CCP in LGL leukemia can be explained by the presence of increased JAK/STAT signaling or activating *STAT3* mutations as observed in our cohort. Further study of this signaling pathway in RA as well as the connection to serological markers in LGL leukemia is warranted.

We propose that the presence of *STAT3* mutations, even in a small percentage of cells, may create differential signaling in RA leading to heightened disease markers such as anti-CCP. A collagen induced arthritis mouse model previously implicated JAK/STAT signaling in disease establishment and showed *STAT3* was necessary for arthritis development (8). A recent study connected germline STAT3 gain-of-function mutations to autoimmune disease through oligoclonal accumulation of effector CD8+ T-cells (35). In RA, we hypothesized that auto-reactive CD8+ T-cells, particularly those bearing *STAT3* mutations, kill target cells such as neutrophils that contain peptidylarginine deiminases (PADs), the enzymes that catalyze citrullination of proteins. Through target cell killing, PADs are activated, leading to protein hypercitrullination. The citrullinated antigens are released as the targeted cells die, inducing wider production of anti-citrullinated protein antibodies (ACPAs) (6). Interestingly, anti-CCP+ RA has also been associated with proliferation and clonal expansion of CD8+ T-cells in response to citrullinated antigens (23, 36), potentially creating a positive feedback loop between *STAT3* mutation, effector CD8+ T-cell accumulation, hypercitrullination, and increased serum anti-CCP positivity. Thus, in our study, the effect of *STAT3* mutations on CD8+ T-cell function may explain the increased anti-CCP positivity observed in patients with *STAT3* mutations. Additionally, a hypercitrullinated environment may increase proliferation of CD8+ T-cells and thus foster conditions in which mutations are more likely to occur or expand. However, mechanistic and biological investigations of the functional consequences of the observed *STAT3* mutations were beyond the scope of this study and were not feasible due to limited yield of PBMC CD8+ cells and no access to synovial CD8+ cells.

Overall, our observations could eventually inform treatment options. For example, rituximab is more effective in RF and anti-CCP-positive RA patients (1, 37, 38). One study saw a 100% response rate to rituximab in LGL leukemia patients with concomitant RA, and there have been several other case reports documenting similar success (39–42). This suggests that *STAT3* mutations may be an indicator for rituximab treatment. At the close of recruitment for our study in 2017, only 2/98 patients had taken rituximab. Therefore, further study is necessary to gauge the relationship between *STAT3* mutation and rituximab efficacy.

Additionally, JAK/STAT signaling inhibition with jakinibs is a rapidly evolving treatment alternative in RA. A recent systematic review showed that upadacitinib and tofacitinib are among the best performers in RA (43). Jakinibs such as ruxolitinib and tofacitinib have also been used to treat LGL leukemia patients with favorable response (44, 45), including a cohort of treatment-refractory patients with both RA and LGL leukemia in which many showed hematological improvement with treatment (46). A recent study of LGL leukemia patients treated with ruxolitinib reported that *STAT3* mutations were predictive of improved event-free survival with 100% of *STAT3* mutant individuals exhibiting event-free survival at 14 months compared to 40% with WT *STAT3* (45). The field of JAK/STAT inhibition in RA is still in its early stages, but *STAT3* mutational profiling in RA patients may provide more informed and effective treatment decisions.

In conclusion, we identified 3% of RA patients with high VAF *STAT3* mutations indicative of a *STAT3* mutant CD8+ T-cell clonal population. Overall, RA patients exhibited substantially more low VAF (<0.05%) mutations as compared to healthy donors. *STAT3* mutations at VAF >0.05% were strongly associated with seropositivity for RF and anti-CCP positivity. Altogether, further study of *STAT3* mutational status has the potential to inform on disease mechanism and treatment options in RA.

## Data Availability

The raw data supporting the conclusions of this article will be made available by the authors, without undue reservation. The code used for statistical analysis can be found here: https://github.com/loughran/RA-STAT3-Manuscript-Statistics.

## Author disclaimer

This work was presented as a Poster Tour Presentation at EULAR 2023 under Moosic K, Olson T, Freijat M, et al. POS0348 ACTIVATING *STAT3* MUTATIONS IN CD8+ T CELLS CORRELATE TO SEROLOGICAL POSITIVITY IN RHEUMATOID ARTHRITIS PATIENTS Annals of the Rheumatic Diseases 2023;82:423-424. Preliminary results from this study were also published in the November 2022 online supplement for the ASH 2022 annual meeting in the journal Blood. Katharine B. Moosic, Thomas L. Olson, Mark Freijat, Samara Khalique, Cait E. Hamele, Bryna Shemo, Jesse Boodoo, William Baker, Matthew W. Schmachtenberg, Erika Darrah, Felipe Andrade, Kristine C. Olson, David J. Feith, Donald L. Kimpel, Thomas P. Loughran; Presence of Activating *STAT3* Mutations in the CD8+ T Cells of Rheumatoid Arthritis Patients. Blood 2022; 140 (Supplement 1): 11192–11193. doi: https://doi.org/10.1182/blood-2022-168069.
