# Supplementary figures and images for: Activating STAT3 mutations in CD8+ T-cells correlate to serological positivity in rheumatoid arthritis
Source: Front Immunol. 2024 Oct 21;15:1466276. doi: 10.3389/fimmu.2024.1466276 (PMC11532115; doi:10.3389/fimmu.2024.1466276)

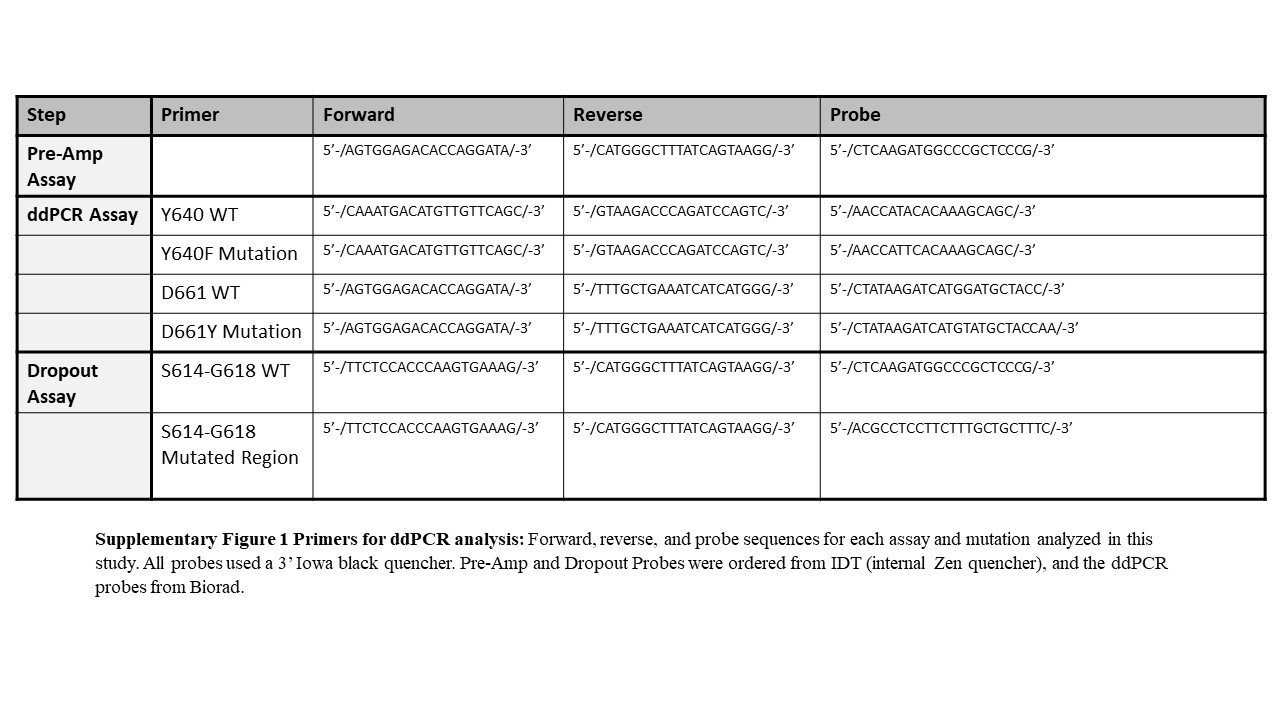

Supplement: Supplementary file 1 [file Image1.jpeg]

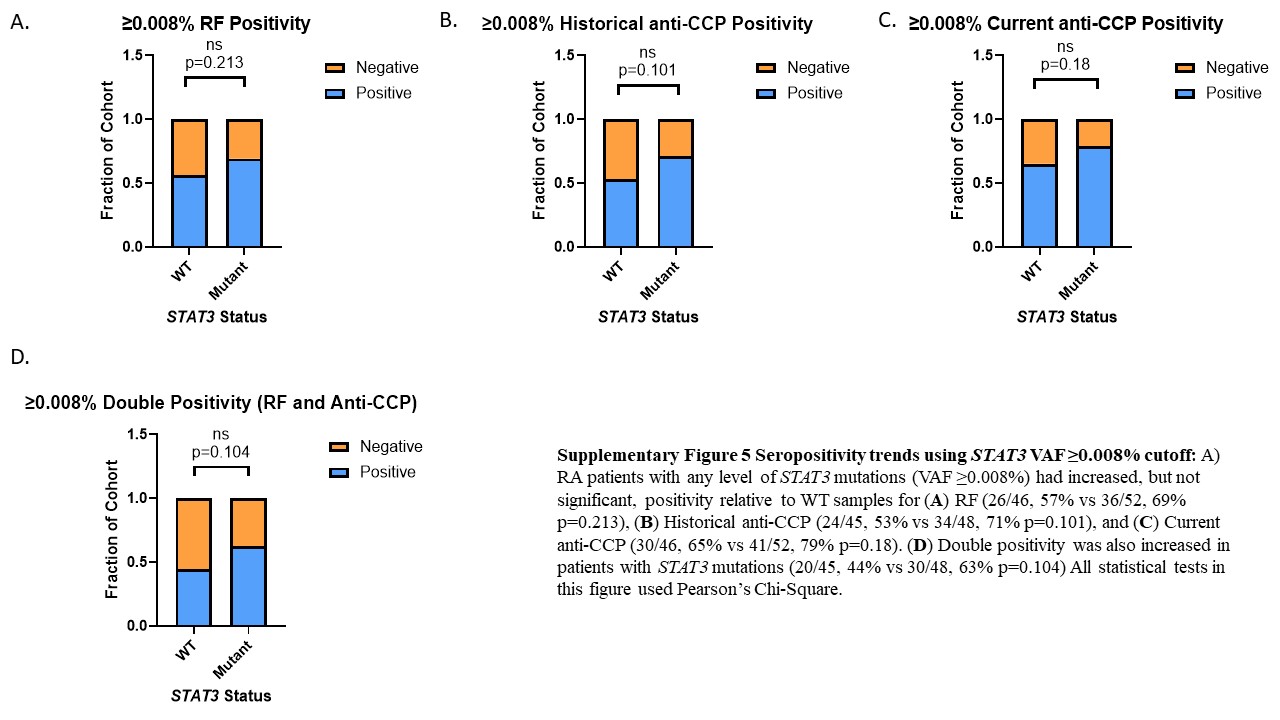

Supplement: Supplementary file 2 [file Image2.jpeg]

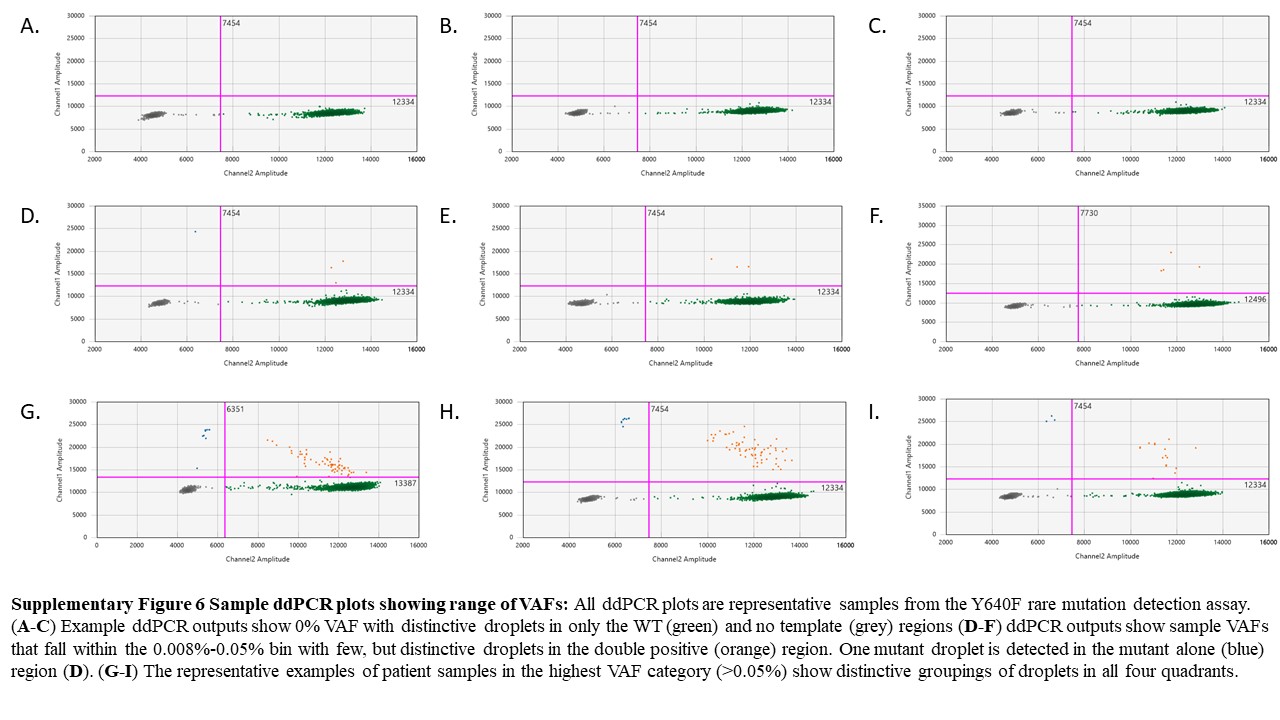

Supplement: Supplementary file 3 [file Image3.jpeg]

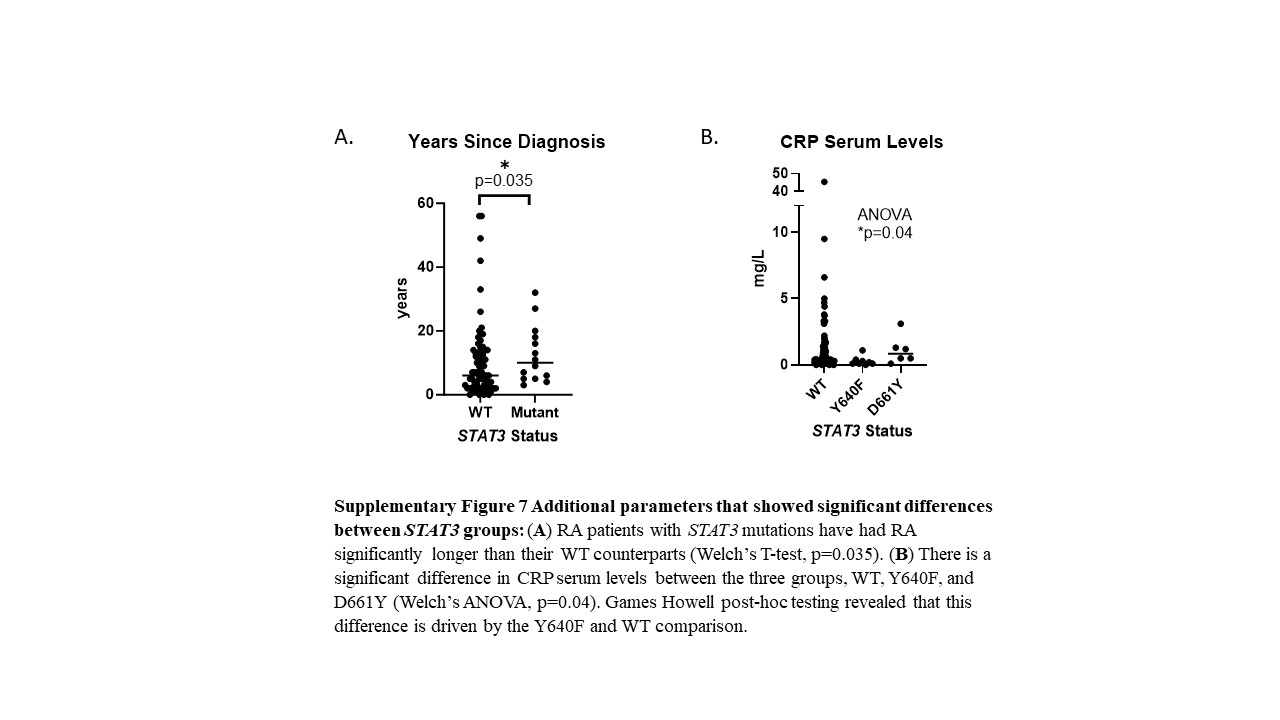

Supplement: Supplementary file 4 [file Image4.jpeg]

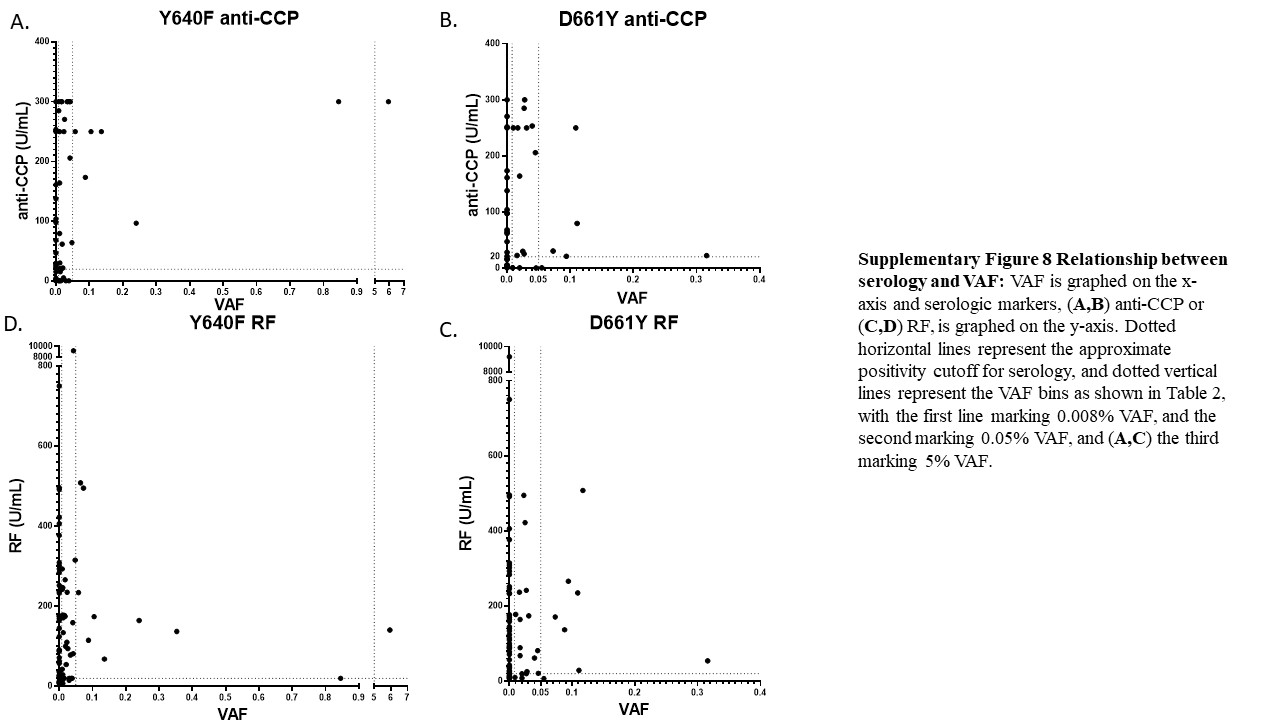

Supplement: Supplementary file 5 [file Image5.jpeg]
